# Supplementary material for: Causal Responsibility Based Explainable AI for Vibrational Spectroscopy Applied to Oral FTIR and Oesophageal Raman Diagnostics
Source: Res Sq. 2026 Jun 29:rs.3.rs-8338382. Preprint. [Version 1] doi: 10.21203/rs.3.rs-8338382/v1 (PMC13345556; doi:10.21203/rs.3.rs-8338382/v1)
Supplement: 1 [file NIHPPRS8338382V1-supplement-1.pdf]

Supplementary information.

A ReX algorithm

Pseudocode for the ReX algorithm for computing responsibility.

Algorithm 1 Responsibility of a spectral interval w.r.t. a partition Π_i

Require: Spectrum x, classifier N, predicted class $o = N(x)$, partition $\Pi_i = \{I_{i,1}, \ldots, I_{i,s}\}$, masking operator $\text{mask}(x, S)$

for $j \leftarrow 1$ to s **do**
  $r_j \leftarrow 0$
  if $N(\text{mask}(x, \{I_{i,j}\})) \neq o$ **then** ▷ Interval alone flips the class
    $r_j \leftarrow 1$; **continue**
  end if
  for $k \leftarrow 1$ to $s - 1$ **do** ▷ Search witness size
    for all $W \subseteq \Pi_i \setminus \{I_{i,j}\}$ with $|W| = k$ **do**
      if $N(\text{mask}(x, W)) = o$ **and** $N(\text{mask}(x, W \cup \{I_{i,j}\})) \neq o$ **then**
        $r_j \leftarrow 1/(k+1)$; **break**
      end if
    end for
    if $r_j > 0$ **then break**
    end if
  end for
end for
return $\{r(I_{i,j}, x, o) = r_j\}_{j=1}^{s}$

B Library Spectra

Spectral data of pure biochemical samples were acquired using a Renishaw inVia Qontor Raman microscope equipped with a 785nm laser and a 50x objective lens (Renishaw plc, Wotton-under-edge, UK). An integration time of 10 seconds was used for all measurements, with each spectral acquisition comprising 10 accumulations per point. All biomolecules used were obtained from Sigma Aldrich, UK.

C *In silico* definitive ground truth results

These results display the full results of the *In silico*: definitive ground truth dataset. Note Class 0, the null class, is defined by the lack of features. This presents a unique challenge for explainability in which post-hoc XAI methods identify features which if present contributes (and in the case of SHAP if present harms) to a classification. However, the null class is defined by the absence of any such features. In Spec-ReX, the identification of the edges of the spectra is a typical hallmark of how the algorithm behaves when a class is defined by the absence of features. Similar behaviour is seen

is ReX with images (with responsibility accruing in a corner). This is a consequence of the heuristics of ReX.

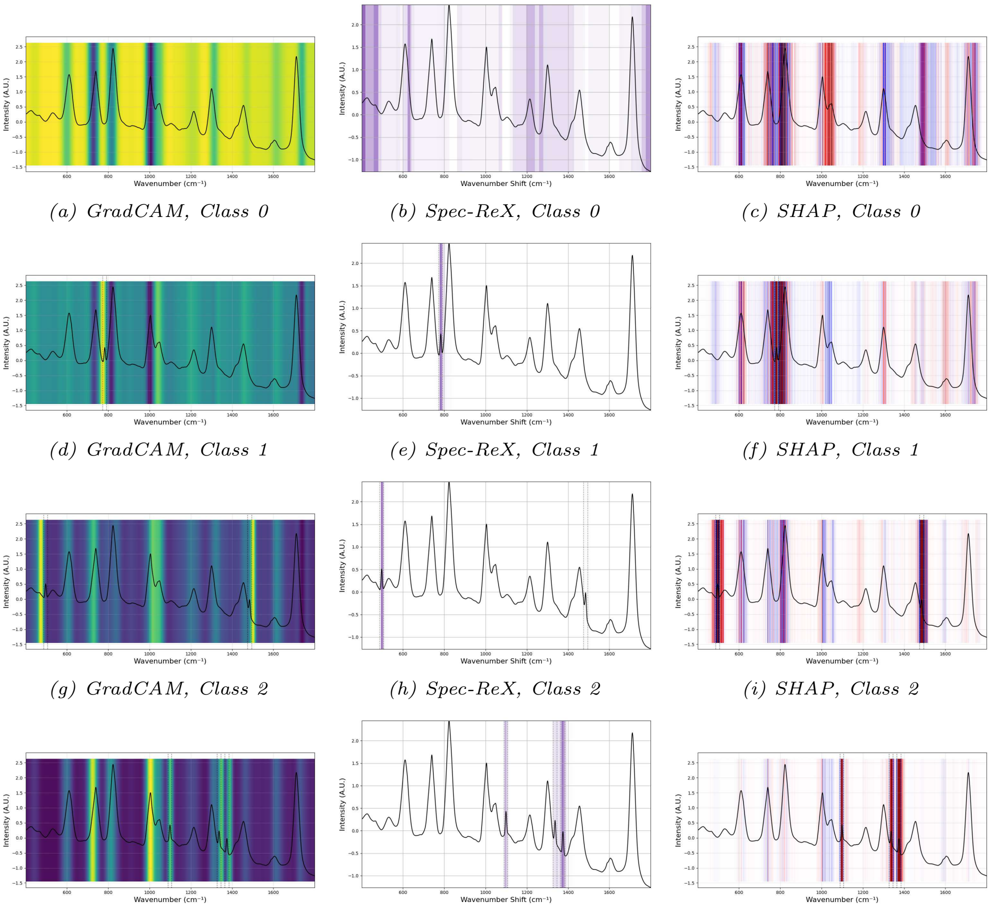

(a) GradCAM, Class 0 *(b) Spec-ReX, Class 0* *(c) SHAP, Class 0*

(d) GradCAM, Class 1 *(e) Spec-ReX, Class 1* *(f) SHAP, Class 1*

(g) GradCAM, Class 2 *(h) Spec-ReX, Class 2* *(i) SHAP, Class 2*

(j) GradCAM, Class 3 *(k) Spec-ReX, Class 3* *(l) SHAP, Class 3*

Fig. C.5 *In silico*: unambiguous ground truth full results of attribution maps by method and class.

Table 7 shows all performance measures, broken down by class. Of note is that the FFid scores generally decrease with the higher class. In this instance the class refers to the number of class discriminant features (i.e. class 3 has 3 features unique to it). The fact the FFid scores lower when there are more discriminative features in consistent with how FFid works. It masks regions deemed important and then fine-tunes the model. This gives the model a chance to relearn new features. Class 1 sees the biggest drop in performance because if the sole discriminating feature is removed, then there is no remaining true signal left, whereas in classes with more than one discriminating feature the model can just identify another feature. Hence, the FFid scores are generally much lower when there are more features present. This would also be the case with ROAR. This shows that such methods are not well suited to cases

Method	Level	IoU	Concentration	iAUC	dAUC	FFid$^+$	FFid$^-$
GradSHAP-meanSpectrum	Overall	0.09 [0.09, 0.09]	0.18 [0.17, 0.18]	0.25 [0.25, 0.26]	0.20 [0.20, 0.20]	0.0595 [0.0592, 0.0599]	0.0616 [0.0609, 0.0623]
	Class 0	0.00 [0.00, 0.00]	0.00 [0.00, 0.00]	0.09 [0.09, 0.09]	0.10 [0.09, 0.10]	0.1502 [0.1476, 0.1528]	0.2453 [0.2416, 0.2490]
	Class 1	0.06 [0.06, 0.06]	0.18 [0.18, 0.19]	0.50 [0.49, 0.51]	0.41 [0.41, 0.42]	0.0883 [0.0876, 0.0891]	0.0033 [0.0030, 0.0037]
	Class 2	0.12 [0.12, 0.12]	0.25 [0.25, 0.26]	0.33 [0.32, 0.34]	0.23 [0.22, 0.23]	**0.0035 [0.0034, 0.0036]**	0.0007 [0.0006, 0.0007]
	Class 3	0.17 [0.17, 0.17]	0.27 [0.27, 0.28]	0.09 [0.09, 0.09]	0.06 [0.05, 0.06]	0.0005 [0.0005, 0.0005]	0.0001 [0.0001, 0.0001]
GradSHAP-randContraLabel	Overall	0.09 [0.09, 0.10]	0.24 [0.24, 0.24]	0.22 [0.21, 0.22]	**0.16 [0.15, 0.16]**	**0.1658 [0.1652, 0.1664]**	0.1435 [0.1427, 0.1442]
	Class 0	0.00 [0.00, 0.00]	0.00 [0.00, 0.00]	0.09 [0.09, 0.09]	0.09 [0.09, 0.09]	**0.5810 [0.5770, 0.5850]**	0.5718 [0.5676, 0.5760]
	Class 1	0.06 [0.06, 0.06]	0.21 [0.21, 0.22]	0.41 [0.40, 0.41]	0.34 [0.34, 0.35]	0.0818 [0.0806, 0.0829]	0.0032 [0.0032, 0.0033]
	Class 2	0.13 [0.13, 0.13]	0.34 [0.33, 0.34]	0.30 [0.29, 0.31]	0.16 [0.16, 0.17]	0.0011 [0.0011, 0.0012]	0.0009 [0.0009, 0.0009]
	Class 3	0.19 [0.19, 0.19]	0.41 [0.40, 0.41]	0.07 [0.07, 0.07]	**0.03 [0.03, 0.03]**	0.0035 [0.0034, 0.0036]	0.0007 [0.0006, 0.0007]
GradSHAP-datasetAvg	Overall	0.09 [0.09, 0.09]	0.26 [0.25, 0.26]	0.22 [0.21, 0.22]	0.16 [0.16, 0.17]	0.1633 [0.1627, 0.1639]	0.1519 [0.1512, 0.1527]
	Class 0	0.00 [0.00, 0.00]	0.00 [0.00, 0.00]	0.10 [0.10, 0.10]	0.10 [0.10, 0.10]	0.5807 [0.5769, 0.5844]	0.6038 [0.5997, 0.6078]
	Class 1	0.06 [0.06, 0.06]	0.26 [0.25, 0.26]	0.42 [0.42, 0.43]	0.36 [0.36, 0.37]	0.0691 [0.0684, 0.0697]	0.0033 [0.0032, 0.0035]
	Class 2	0.12 [0.12, 0.12]	0.34 [0.34, 0.34]	0.26 [0.26, 0.27]	0.16 [0.15, 0.16]	0.0030 [0.0029, 0.0030]	0.0002 [0.0002, 0.0002]
	Class 3	0.19 [0.19, 0.19]	0.42 [0.42, 0.43]	0.08 [0.08, 0.08]	0.03 [0.03, 0.03]	0.0014 [0.0014, 0.0015]	0.0003 [0.0003, 0.0004]
Grad-CAM	Overall	0.01 [0.01, 0.01]	0.01 [0.01, 0.01]	0.43 [0.42, 0.44]	0.47 [0.46, 0.47]	0.0033 [0.0024, 0.0042]	0.0015 [0.0011, 0.0017]
	Class 0	0.00 [0.00, 0.00]	0.00 [0.00, 0.00]	0.08 [0.07, 0.08]	**0.07 [0.06, 0.07]**	0.0105 [0.0070, 0.0139]	**0.0001 [0.0000, 0.0003]**
	Class 1	0.01 [0.01, 0.01]	0.00 [0.00, 0.00]	0.82 [0.81, 0.82]	0.91 [0.91, 0.91]	-0.0006 [-0.0013, -0.0002]	0.0052 [0.0028, 0.0067]
	Class 2	0.01 [0.01, 0.01]	0.01 [0.01, 0.01]	0.54 [0.53, 0.55]	0.55 [0.54, 0.56]	-0.0001 [-0.0004, 0.0003]	0.0001 [-0.0001, 0.0003]
	Class 3	0.03 [0.03, 0.03]	0.02 [0.02, 0.02]	0.28 [0.28, 0.29]	0.34 [0.33, 0.35]	0.0002 [0.0001, 0.0002]	0.0001 [0.0001, 0.0002]
ReX-quadratic	Overall	**0.10 [0.09, 0.10]**	**0.56 [0.56, 0.57]**	0.70 [0.69, 0.70]	0.16 [0.16, 0.16]	0.0377 [0.0312, 0.0487]	0.0041 [0.0028, 0.0055]
	Class 0	0.00 [0.00, 0.00]	0.00 [0.00, 0.00]	0.32 [0.32, 0.33]	0.27 [0.27, 0.28]	-0.0004 [-0.0005, -0.0004]	0.0159 [0.0118, 0.0205]
	Class 1	**0.06 [0.06, 0.07]**	**0.84 [0.84, 0.85]**	0.88 [0.88, 0.89]	0.18 [0.18, 0.18]	0.1614 [0.1348, 0.2054]	**-0.0031 [-0.0034, -0.0029]**
	Class 2	**0.13 [0.12, 0.13]**	**0.85 [0.84, 0.85]**	0.80 [0.79, 0.80]	**0.11 [0.11, 0.11]**	0.0020 [0.0013, 0.0024]	-0.0002 [-0.0004, -0.0001]
	Class 3	**0.19 [0.19, 0.19]**	0.56 [0.56, 0.57]	0.78 [0.78, 0.78]	0.08 [0.08, 0.08]	**0.0008 [0.0005, 0.0011]**	**0.0000 [0.0000, 0.0001]**
ReX-linear	Overall	0.07 [0.07, 0.07]	0.44 [0.43, 0.44]	0.67 [0.67, 0.68]	0.17 [0.16, 0.17]	0.0398 [0.0318, 0.0527]	**0.0000 [-0.0006, 0.0004]**
	Class 0	0.00 [0.00, 0.00]	0.00 [0.00, 0.00]	0.28 [0.28, 0.28]	0.27 [0.26, 0.27]	0.0005 [0.0000, 0.0010]	0.0037 [0.0010, 0.0056]
	Class 1	0.06 [0.06, 0.06]	0.49 [0.48, 0.50]	0.87 [0.87, 0.87]	**0.16 [0.16, 0.16]**	**0.1722 [0.1377, 0.2261]**	-0.0018 [-0.0019, -0.0016]
	Class 2	0.09 [0.09, 0.09]	0.66 [0.65, 0.67]	0.78 [0.78, 0.79]	0.14 [0.14, 0.14]	0.0018 [0.0013, 0.0024]	0.0003 [0.0001, 0.0005]
	Class 3	0.14 [0.14, 0.14]	**0.60 [0.59, 0.60]**	0.75 [0.75, 0.75]	0.09 [0.09, 0.09]	0.0004 [0.0001, 0.0006]	0.0000 [0.0000, 0.0000]
ReX-min	Overall	0.07 [0.07, 0.08]	0.09 [0.09, 0.10]	**0.76 [0.76, 0.77]**	0.25 [0.25, 0.26]	0.0037 [0.0013, 0.0072]	0.0091 [0.0046, 0.0136]
	Class 0	0.00 [0.00, 0.00]	0.00 [0.00, 0.00]	**0.55 [0.55, 0.56]**	0.49 [0.48, 0.49]	0.0000 [0.0000, 0.0000]	0.0363 [0.0194, 0.0532]
	Class 1	0.06 [0.06, 0.06]	0.04 [0.04, 0.04]	**0.91 [0.90, 0.91]**	0.25 [0.25, 0.26]	0.0214 [0.0088, 0.0387]	-0.0016 [-0.0018, -0.0013]
	Class 2	0.08 [0.08, 0.08]	0.12 [0.11, 0.12]	0.79 [0.78, 0.79]	0.17 [0.16, 0.17]	0.0002 [0.0001, 0.0004]	**-0.0004 [-0.0005, -0.0003]**
	Class 3	0.15 [0.15, 0.15]	0.21 [0.21, 0.22]	**0.81 [0.81, 0.81]**	0.11 [0.11, 0.11]	0.0000 [0.0000, 0.0000]	0.0000 [0.0000, 0.0001]
ReX-mean	Overall	0.07 [0.07, 0.07]	0.22 [0.22, 0.23]	0.72 [0.72, 0.72]	0.20 [0.19, 0.20]	0.0391 [0.0307, 0.0439]	0.0184 [0.0111, 0.0258]
	Class 0	0.00 [0.00, 0.00]	0.00 [0.00, 0.00]	0.41 [0.40, 0.41]	0.34 [0.33, 0.35]	0.0034 [0.0034, 0.0034]	0.0597 [0.0328, 0.0866]
	Class 1	0.06 [0.06, 0.06]	0.27 [0.26, 0.28]	0.88 [0.88, 0.88]	0.17 [0.17, 0.18]	0.1570 [0.1255, 0.1753]	0.0015 [0.0012, 0.0018]
	Class 2	0.08 [0.08, 0.09]	0.26 [0.25, 0.27]	**0.80 [0.80, 0.81]**	0.17 [0.17, 0.17]	0.0027 [0.0019, 0.0032]	0.0011 [0.0008, 0.0014]
	Class 3	0.14 [0.14, 0.14]	0.36 [0.35, 0.36]	0.79 [0.79, 0.79]	0.10 [0.10, 0.10]	0.0001 [0.0000, 0.0001]	0.0004 [0.0002, 0.0005]

Table 7 In Silico definitive ground truth on test set: IoU, Concentration, Insertion AUC (Ins, higher is better), Deletion AUC (Del, lower is better), and F-Fidelity (FFid$^+$ $\uparrow$, FFid$^-$ $\downarrow$). Values are mean [95% CI]. **Bold** marks the best performance for the class.

when there are many correlated features which distinguish a class, as might be the case in vibrational spectroscopy.

Method	Baseline	iAUC (Ins ↑)	dAUC (Del ↓)	iAUC mean (AVG)	dAUC mean (AVG)
GradSHAP-datasetAvg	zero	0.18 [0.18, 0.18]	0.16 [0.16, 0.17]	0.22 [0.21, 0.22]	0.16 [0.16, 0.17]
	datasetAvg	0.22 [0.22, 0.22]	0.16 [0.15, 0.16]		
	randOtherClass	0.24 [0.23, 0.25]	0.17 [0.17, 0.18]		
	meanOtherClass	0.22 [0.22, 0.23]	0.16 [0.15, 0.16]		
GradSHAP-randContraLabel	zero	0.18 [0.18, 0.19]	0.15 [0.15, 0.15]	0.22 [0.21, 0.22]	0.16 [0.15, 0.16]
	datasetAvg	0.22 [0.21, 0.22]	0.16 [0.15, 0.16]		
	randOtherClass	0.24 [0.23, 0.25]	0.16 [0.16, 0.17]		
	meanOtherClass	0.22 [0.21, 0.22]	0.16 [0.15, 0.16]		
GradSHAP-meanSpectrum	zero	0.20 [0.20, 0.20]	0.17 [0.16, 0.17]	0.25 [0.25, 0.26]	0.20 [0.20, 0.20]
	datasetAvg	0.27 [0.26, 0.27]	0.21 [0.21, 0.22]		
	randOtherClass	0.27 [0.27, 0.28]	0.21 [0.20, 0.21]		
	meanOtherClass	0.27 [0.26, 0.27]	0.21 [0.21, 0.21]		
Grad-CAM	zero	0.44 [0.44, 0.45]	0.48 [0.47, 0.49]	0.43 [0.42, 0.44]	0.47 [0.46, 0.47]
	datasetAvg	0.44 [0.44, 0.45]	0.48 [0.47, 0.49]		
	randOtherClass	0.38 [0.37, 0.38]	0.42 [0.41, 0.43]		
	meanOtherClass	0.45 [0.44, 0.46]	0.49 [0.48, 0.49]		
ReX-quadratic	zero	0.70 [0.69, 0.70]	0.15 [0.15, 0.15]	0.70 [0.69, 0.70]	0.16 [0.16, 0.16]
	datasetAvg	0.77 [0.76, 0.77]	0.17 [0.16, 0.17]		
	randOtherClass	0.55 [0.54, 0.56]	0.16 [0.16, 0.17]		
	meanOtherClass	0.77 [0.76, 0.77]	0.16 [0.16, 0.17]		
ReX-linear	zero	0.66 [0.66, 0.67]	0.16 [0.15, 0.16]	0.67 [0.67, 0.68]	0.17 [0.16, 0.17]
	datasetAvg	0.74 [0.74, 0.75]	0.18 [0.17, 0.18]		
	randOtherClass	0.53 [0.53, 0.54]	0.16 [0.16, 0.16]		
	meanOtherClass	0.74 [0.74, 0.75]	0.17 [0.17, 0.18]		
ReX-min	zero	0.79 [0.79, 0.79]	0.26 [0.26, 0.26]	0.76 [0.76, 0.77]	0.25 [0.25, 0.26]
	datasetAvg	0.84 [0.84, 0.85]	0.27 [0.26, 0.27]		
	randOtherClass	0.59 [0.58, 0.59]	0.22 [0.22, 0.22]		
	meanOtherClass	0.84 [0.84, 0.84]	0.26 [0.26, 0.27]		
ReX-mean	zero	0.74 [0.73, 0.74]	0.18 [0.17, 0.18]	0.72 [0.72, 0.72]	0.20 [0.19, 0.20]
	datasetAvg	0.80 [0.80, 0.81]	0.21 [0.21, 0.21]		
	randOtherClass	0.54 [0.53, 0.54]	0.19 [0.19, 0.19]		
	meanOtherClass	0.80 [0.80, 0.80]	0.21 [0.20, 0.21]		

Table 8 Insertion/Deletion on the test set. Values are mean [95% CI]. Baseline-specific iAUC/dAUC are shown; the rightmost columns report the baseline-averaged (mean) iAUC/dAUC for each method.

Insertion/deletion AUC by masking method

Table 8 shows the iAUC and dAUC broken down by masking method, and with the mean of all masking methods (which is what has been reported in the main text). The different masking methods return largely consistent results. The one exception is that Spec-ReX's iAUC suffers in performance when a random spectrum from the other class is used to provide the masking values. This will slightly bring down Spec-Rex's average iAUC performance. This is what has been used throughout all other experiments.

D *In Silico*: ambiguous ground truth results

Method	Level	IoU	PHR	iAUC	dAUC	FFid^{+}	FFid^{-}
GradientSHAP	Overall			**0.84 [0.83, 0.85]**	**0.52 [0.51, 0.52]**	**0.0006 [0.0002, 0.0010]**	0.0093 [0.0073, 0.0114]
	Class 0			**0.95 [0.95, 0.96]**	**0.56 [0.55, 0.57]**	**0.0004 [0.0003, 0.0004]**	0.0014 [0.0012, 0.0018]
	Class 1	0.04 [0.04, 0.04]	**0.31 [0.31, 0.32]**	0.73 [0.72, 0.73]	**0.48 [0.47, 0.48]**	**0.0019 [0.0011, 0.0028]**	0.0183 [0.0145, 0.0222]
Grad-CAM	Overall			0.66 [0.65, 0.66]	0.69 [0.68, 0.69]	0.0003 [0.0003, 0.0003]	0.0033 [0.0025, 0.0038]
	Class 0			0.63 [0.62, 0.63]	0.60 [0.60, 0.61]	0.0003 [0.0002, 0.0004]	0.0039 [0.0037, 0.0040]
	Class 1	0.02 [0.02, 0.02]	0.11 [0.11, 0.12]	0.76 [0.75, 0.76]	0.85 [0.85, 0.86]	-0.0001 [-0.0003, 0.0001]	0.0021 [0.0007, 0.0030]
Spec-ReX	Overall			0.74 [0.73, 0.74]	0.58 [0.58, 0.59]	0.0003 [0.0002, 0.0004]	**-0.0000 [-0.0001, 0.0000]**
	Class 0			0.58 [0.58, 0.59]	0.59 [0.58, 0.60]	-0.0005 [-0.0006, -0.0004]	**-0.0007 [-0.0008, -0.0006]**
	Class 1	**0.05 [0.05, 0.05]**	0.24 [0.24, 0.25]	**0.85 [0.84, 0.85]**	0.58 [0.57, 0.58]	0.0012 [0.0009, 0.0016]	**0.0007 [0.0006, 0.0008]**

Table 9 Ambiguous In Silico: IoU, Point Hit Rate (PHR), Insertion AUC (Ins, ↑), Deletion AUC (Del, ↓), and F-Fidelity (FFid^{+} ↑, FFid^{-} ↓). All results are means [95% CI].

E *In Vitro* Experiment Full Results

Method	Level	IoU	PHR	iAUC	dAUC	$FFid^{+}$	$FFid^{-}$
GradientSHAP	Overall	0.0000 [0.0000, 0.0000]	0.0000 [0.0000, 0.0000]	0.48 [0.47, 0.49]	**0.38 [0.37, 0.39]**	**0.0470 [0.0420, 0.0522]**	0.0322 [0.0287, 0.0361]
	Class 0			**0.95 [0.95, 0.95]**	0.83 [0.83, 0.83]	**0.1181 [0.1078, 0.1284]**	**-0.0295 [-0.0309, -0.0280]**
	Class 1			0.11 [0.10, 0.11]	**0.05 [0.05, 0.05]**	-0.0019 [-0.0056, 0.0021]	0.1147 [0.1044, 0.1266]
	Class 2			0.28 [0.27, 0.28]	**0.14 [0.14, 0.14]**	**0.0133 [0.0116, 0.0152]**	0.0011 [0.0008, 0.0014]
Grad-CAM	Overall	**0.0094 [0.0091, 0.0097]**	**0.3371 [0.3264, 0.3487]**	0.52 [0.51, 0.53]	0.59 [0.58, 0.59]	0.0129 [0.0059, 0.0188]	0.0031 [0.0018, 0.0043]
	Class 0			0.75 [0.74, 0.75]	0.71 [0.70, 0.72]	0.0497 [0.0296, 0.0676]	0.0066 [0.0060, 0.0073]
	Class 1			0.17 [0.16, 0.18]	0.36 [0.35, 0.37]	-0.0117 [-0.0146, -0.0088]	0.0064 [0.0043, 0.0086]
	Class 2			0.75 [0.75, 0.76]	0.79 [0.78, 0.80]	0.0015 [0.0007, 0.0019]	**0.0005 [0.0002, 0.0008]**
Spec-ReX	Overall	0.0008 [0.0007, 0.0010]	0.0221 [0.0179, 0.0262]	**0.63 [0.62, 0.64]**	0.41 [0.41, 0.42]	0.0037 [0.0022, 0.0049]	**0.0007 [0.0001, 0.0012]**
	Class 0			0.81 [0.81, 0.82]	**0.52 [0.51, 0.52]**	0.0101 [0.0090, 0.0113]	0.0031 [0.0027, 0.0034]
	Class 1			**0.38 [0.36, 0.39]**	0.19 [0.18, 0.19]	**0.0048 [0.0022, 0.0075]**	**0.0024 [0.0017, 0.0029]**
	Class 2			**0.78 [0.77, 0.79]**	0.66 [0.66, 0.67]	0.0003 [0.0001, 0.0005]	0.0069 [0.0050, 0.0080]

Table 10 *In Vitro* performance vs DNA peaks: IoU (↑), PHR (↑); iAUC (↑), dAUC (↓); $FFid^{+}$ (↑), $FFid^{-}$ (↓). Values are mean [95% CI].

F Ex Vivo, Oesaphageal Cancer Results for the ResNet

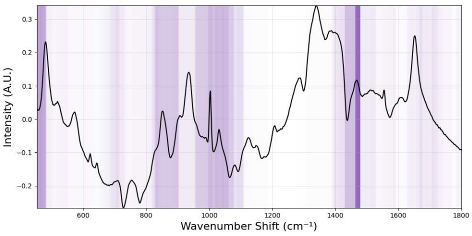

(a) ReX, NSQ

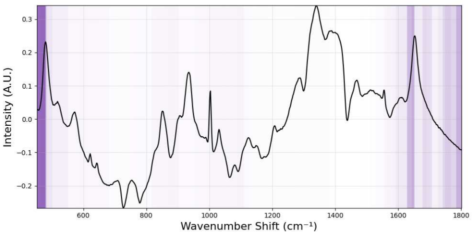

(d) ReX, AC

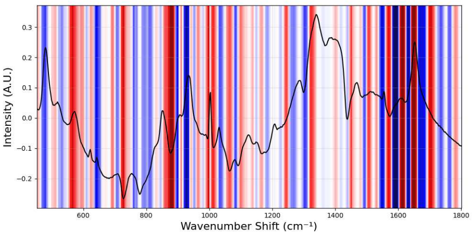

(b) SHAP, NSQ

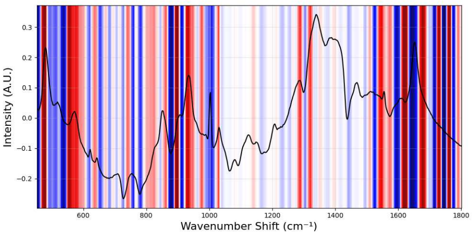

(e) SHAP, AC

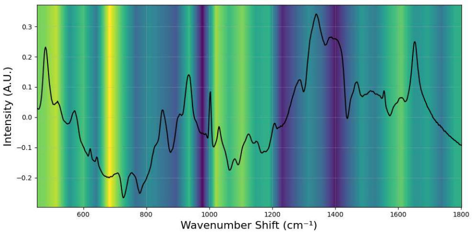

(c) Grad-CAM, NSQ

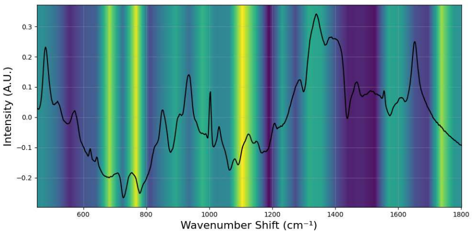

(f) Grad-CAM, AC

Fig. F.6 Ex Vivo Results, Oesaphageal Cancer, ResNet. NSQ vs HGD and AC. Difference spectra and mean attribution maps arranged by method (rows) and class (columns).

F.1 Ex Vivo, Oesaphageal Cancer Results for the Custom CNN

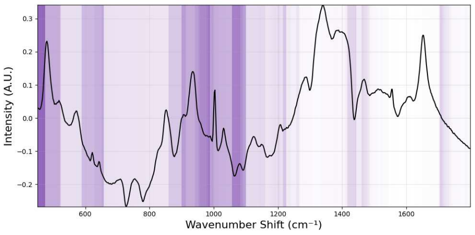

(a) ReX, NSQ

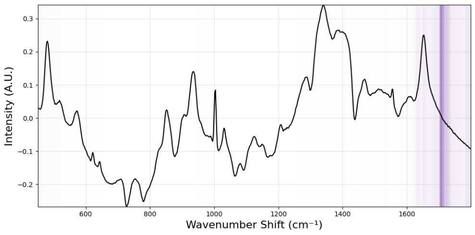

(d) ReX, AC

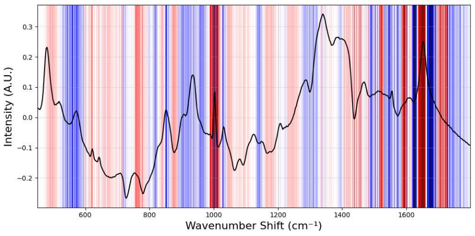

(b) SHAP, NSQ

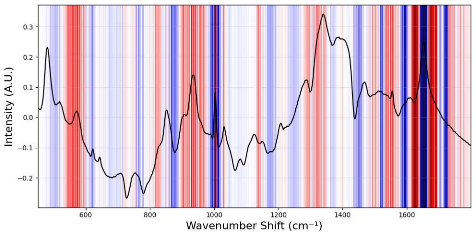

(e) SHAP, AC

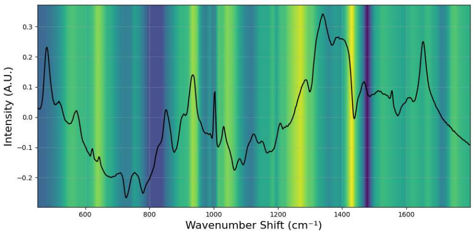

(c) Grad-CAM, NSQ

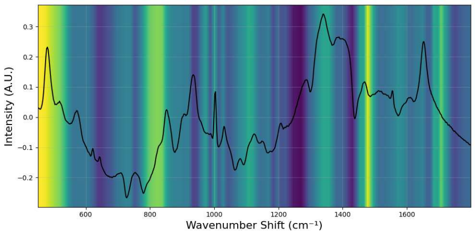

(f) Grad-CAM, AC

Fig. F.7 Ex Vivo Results, Oesaphageal Cancer, Custom CNN. NSQ vs HGD and AC. Difference spectra and mean attribution maps arranged by method (rows) and class (columns).

F.2 Ex Vivo, FTIR Oral Cancer Results

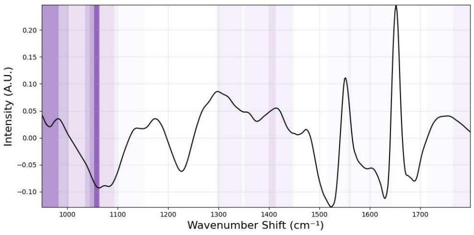

(a) ReX, Benign

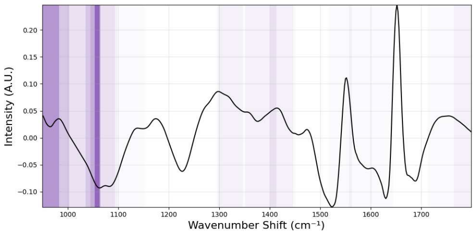

(d) ReX, Malignant

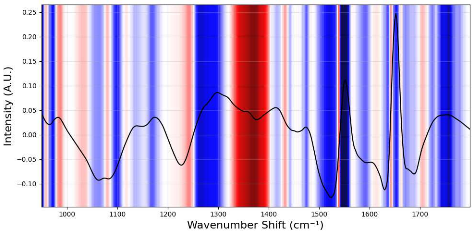

(b) SHAP, Benign

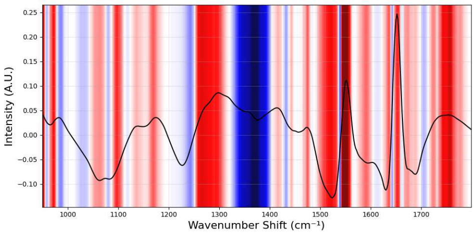

(e) SHAP, Malignant

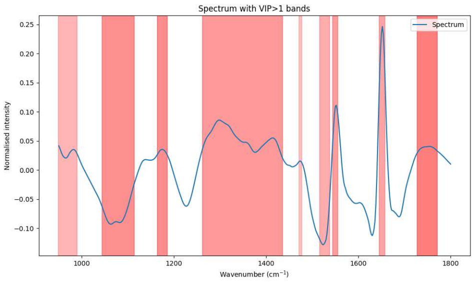

(c) VIP, Benign

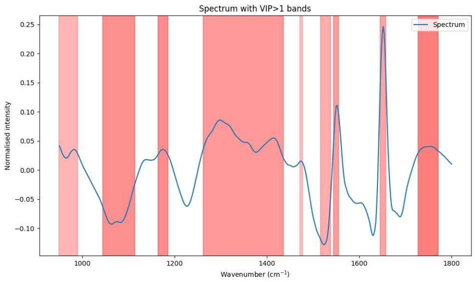

(f) VIP, Malignant

Fig. F.8 Difference spectra and mean attribution maps arranged by method (rows) and class (columns).
